# Immunological changes associated with the treatment of pneumonic tularaemia in marmosets

**DOI:** 10.3389/fcimb.2026.1781926

**Published:** 2026-04-30

**Authors:** Rachel E. Ireland, Alejandro Nunez, James D. Blanchard, Francis Dayton, Igor Gonda, Sarah V. Harding, Michelle Nelson

**Affiliations:** 1 Human and Biological Advantages, Defence Science and technology Laboratory, Salisbury, Wiltshire, United Kingdom; 2 Pathology Department, Animal and Plant Health Agency, Addlestone, Surrey, United Kingdom; 3 Formerly employee (at the time this research was conducted) of Aradigm Corporation, Hayward, CA, United States

**Keywords:** aerosol infection, ciprofloxacin, *Francisella tularensis*, innate immunity, interferon-gamma (IFN-γ), non-human primate model, γδ T-cells

## Abstract

**Introduction:**

*Francisella tularensis* is the causative agent of tularemia, a severe infection that can be fatal if untreated. There is a need for improved therapeutic options to enhance the efficacy of existing antibiotic regimens for severe tularemia and to reduce the risk of disease relapse. Novel formulations of ciprofloxacin have demonstrated protection against inhalational tularemia in murine and marmoset models; however, a detailed immunological characterisation of treatment response is required.

**Methods:**

Blood samples were collected from marmosets infected with a lethal dose of inhalational tularemia and treated with either oral ciprofloxacin or inhaled ciprofloxacin (Apulmiq®). Immunological responses were assessed longitudinally and compared with immunological changes previously observed in recovering human cases of tularemia.

**Results:**

Infected marmosets exhibited increased neutrophil levels, which, together with reduced HLA-DR expression, indicated severe infection. Rising circulating interferon-gamma (IFN γ) levels were followed by classical activation of macrophages as an initial attempt to control bacterial dissemination. Progressive disease was characterised by a marked depletion of neutrophils, excessive IFN γ production, and elevated levels of overactivated macrophages. In animals that responded successfully to treatment, these immunological parameters returned to baseline levels. Recovery was subsequently associated with increased T cell populations, particularly gamma delta T (γδ T) cells, and limited antibody production, suggesting the development of long-term immunity comparable to that observed in humans.

**Discussion:**

The observed immunological changes in marmosets closely mirror those reported in human tularemia, supporting the relevance of this model for studying disease progression and recovery. The capacity to monitor these immune parameters demonstrates the utility of the marmoset model in assessing the efficacy of new and novel antibiotic treatments for tularemia.

## 1 Introduction

*Francisella tularensis* is the causative agent of the disease tularemia, which has a low infectious dose and causes an acute fever and can be fatal if untreated (Nelson et al., 2024). Tularemia is endemic in the Northern hemisphere, particularly Northern Europe, Turkey, and the United States (Nelson et al., 2025. Disease is primarily transmitted by arthropod bites, by inhalation through contact with infected animals, typically rabbits or squirrels, or through ingestion (Dennis, 2001 #558). Tularemia is currently treatable with antibiotics such as gentamicin, doxycycline, or ciprofloxacin (Maurin et al., 2023). Ciprofloxacin, doxycycline, levofloxacin, and gentamicin are now recommended by the CDC as first-line antimicrobials (Nelson et al., 2025). However, concerns over the potential for relapse means there is interest in exploring alternative treatments and/or delivery strategies (Caspar and Maurin, 2017). Well-characterised, appropriate animal models that mimic human disease both pathologically and immunologically are critical to this evaluation (Guina et al., 2018; Maurin et al., 2023).

The immunological response to *F. tularensis* in mice has been extensively studied (Fritz et al., 2014; Conlan et al., 2002; Fortier et al., 1991). The initial innate response is composed of both neutrophils and more predominantly macrophages (Roberts et al., 2014). The role of neutrophils is controversial, as *F. tularensis* is known to be able to replicate inside neutrophils *in vitro* (Kinkead and Allen, 2016; McCaffrey and Allen, 2006). *In vivo* IFN-γ recruits significant numbers of neutrophils, which have been shown to be essential for survival (Sjöstedt et al., 1994). The role of macrophages is better understood; *F. tularensis* avoids phagocytic degradation when engulfed by unstimulated macrophages (Clemens et al., 2004). However, IFN-γ-stimulated macrophages (termed classically activated) are resistant to infection and able to kill intracellular bacteria (Steiner et al., 2017) (Fortier et al., 1992; Jessop et al., 2020). *F. tularensis* LPS has an atypical structure, which largely prevents its recognition by endotoxic binding structures, thus delaying macrophage activation (Soni et al., 2010; Hartley et al., 2006; Rowe and Huntley, 2015). Failure of the initial infection to initiate sufficient levels of pro-inflammatory cytokines quickly is thought to result in significant disease (Elkins et al., 2007; Cowley, 2009). Adaptive long-lasting immunity is provided by both T and B cells (Kirimanjeswara et al., 2008; Cole et al., 2009; De Pascalis et al., 2015). Antibodies are known to play a role in protection (Chandler et al., 2015; Kubelkova et al., 2021; Mara-Koosham et al., 2011). Clearance of *F. tularensis* is thought to be due to CD8^+^ effector T cells producing IFN-γ (and TNF-α), activating macrophages, and leading to the production of toxic reactive oxygen and nitrogen molecules, in addition to classical cytotoxic killing by CD8^+^ T cells (Griffin et al., 2015; Elkins et al., 2007).

The immunological response in mice is very different to the human response in a number of important ways. This is demonstrated by the fatal disease, in mice, caused by the live attenuated vaccine strain (LVS) (Griffin et al., 2015). The antibody response in humans is predominately directed towards the LPS rather than towards proteins (Rahhal et al., 2007; Eyles et al., 2007). Human recovery from tularemia is associated with a significant expansion of a specific class of the circulating gamma delta T (γδT) cell (Chrdle et al., 2019)—a cell type that is absent in mice (Heilig and Tonegawa, 1986)—population. Thus, there is a requirement for a more immunologically relevant model of tularemia in a species more closely related to humans, such as non-human primates (NHP).

Two NHP models of tularemia have been described, the common marmoset (Nelson et al., 2010b, 2010a, 2009) and the cynomolgus macaque (Glynn et al., 2015; Guina et al., 2018; Frick et al., 2021; Williams, 2024), sharing key characteristics with human disease. In addition, the cynomolgus macaque model has been fully approved by the FDA under the Animal Model Qualification program (https://force-dsc.my.site.com/ddt/s/ddt-project?ddtprojectid=145). The pathogenesis of disease in this model is well documented, including observed increases in the circulating neutrophils associated with disease; however, the immunological correlates of protection are not described. Marmoset immunology (especially in response to infection) is an evolving field and the ability to measure immunological changes with respect to disease has improved, providing greater clarity in the assessment of the efficacy of medical countermeasures (Nelson and Loveday, 2014; Ngugi et al., 2022).

Previous work describes the survival of marmosets treated with orally or inhalationally administered ciprofloxacin following challenge with *F. tularensis* strain Schu S4 by the aerosol route (Ireland et al., 2026). Animals were challenged with a lethal dose of aerosolised *F. tularensis* and treated either as post-exposure prophylaxis (PEP, 24 h post-challenge) or on the development of fever (Tx, between 57 and 70 h post-challenge) with either oral ciprofloxacin or inhaled Apulmiq or placebo (performed as two independent studies). All placebo-treated animals succumbed to infection between 4.8 and 7.7 days, whereas all treated animals resolved their fever and recovered. However, one animal that received Apulmiq at the onset of fever relapsed with tularemia. This animal developed a secondary fever initiated on day 13 post-challenge despite the absence of other clinical signs of disease. This animal was also the only animal that was colonised with bacteria in their lungs 2 weeks following the cessation of antibiotic treatment.

The aim of this current work is to elucidate the immunological response using samples taken from the animals previously administered with oral or inhalational ciprofloxacin, to determine whether any key immunological features could be associated with effective treatment, survival, or potential relapse.

## 2 Materials and methods

### 2.1 Animals

All immunological data was generated from samples collected in a previously reported study which determined the level of protection offered to marmosets treated with orally or inhalationally administered ciprofloxacin following challenge with aerosolised *F. tularensis* (Ireland et al., 2026). Full experimental details are provided in that manuscript; however, brief details are included here for completeness.

Healthy, sexually mature common marmosets (*Callithrix jacchus*) were obtained from the Dstl Porton Down breeding colony and housed in female and vasectomised male pairs. Animals were aged between 1.4 and 5 years old and weighed between 353 and 498 g at the start of the study. Prior to use in the efficacy studies, all animals were surgically implanted intraperitoneally (i.p.), under general anaesthesia with a Remo 201 device (EMMS Bordon, Hampshire, UK) to record the core body temperature (Tc) remotely. For surgery, animals were initially sedated with a mixture of 3 mg of ketamine hydrochloride (0.03 mL of 100 mg/mL solution) and 30 mg of medetomidine hydrochloride (0.03 mL of 1 mg/mL solution) by the intramuscular route. They were then maintained under 0.5%–3% isoflurane in O_2_ via face mask. Prophylactic pain relief consisting of 0.2 mg/kg of meloxicam and 0.005 mg/kg of buprenorphine was administered. At least 7 days prior to challenge, blood was collected from all animals to assess the baseline levels of immunological markers. Animals were challenged with aerosols of *F. tularensis* which were generated using a 3-jet Collison nebuliser and conditioned using an AeroMP (Aerosol Management Platform) aerosol system (Biaera Technologies L.L.C.). Two cohorts of animals were used; for the PEP study, a cohort of 16 animals were randomly allocated, in pairs, to four groups (no treatment, empty liposomes, Apulmiq, and oral ciprofloxacin). For the Tx study, a cohort of 12 animals were randomly allocated, in pairs, to three groups (Apulmiq, oral ciprofloxacin, and placebo). Animals were administered antibiotics either 24 h post-challenge (PEP) or at the onset of fever (Tx). Inhalational ciprofloxacin (Apulmiq) was delivered by the inhalational route to give a lung dose of 0.8 mg/kg once a day for 7 days and ciprofloxacin (50 mg/kg) was administered orally twice daily for 7 days. The control animals received either empty liposomes delivered by the inhalational route once a day for 7 days, or placebo (banana-flavoured Nesquik^®^ powder reconstituted in distilled water) administered via the oral route twice a day for 7 days. Animals that succumbed to disease and those that survived until the end of the study were humanely euthanised; initially, they were sedated with 0.1 mL of ketamine (100 mg/mL solution) by the intramuscular route, prior to be given an overdose (1–2 mL) of sodium pentobarbital (200 mg/mL solution) by the intraperitoneal route. Blood and tissues (liver, spleen, kidney, and lungs) were then collected for further analysis.

### 2.2 Blood sample time points

Blood was collected from the femoral vein (in-life sampling) of all animals pre-challenge to assess the baseline levels of all immunological markers. In addition, animals that received ciprofloxacin at the onset of fever (Tx) had blood collected at this point (before antibiotic administration) on day 3, at the end of the treatment (day 10 post-challenge) and on day 17. At the time of euthanasia, due either to animals reaching the humane endpoint or at the end of the study (day 21 or day 24), blood was collected by cardiac puncture once the animal was euthanised.

### 2.3 Cell phenotyping

Single-cell suspensions of blood (post red cell lysis) were collected and stained using fluorescent anti-human antibody sets to identify the cell phenotypes and activation status by flow cytometry. The antibodies used to detect lymphocytes were CD3 (SP34-2), CD8 (LT8), CD56 (B159), CD69 (FN50), TCRgd (B1), CD20 (Bly1), and CD16 (3G8), and those for monocytes/macrophages and neutrophils were CD11c (SHCL3), CD14 (M5E2), CD16 (3G8), CD40 (5C3), CD80 (2D10), CD54 (HCD54), CD163 (GHI/61), and HLA-DR (L243) (BD Bioscience, BioLegend, AbD Serotec). Stained cells were fixed for 36 h in a final volume of 4% paraformaldehyde. Samples were analysed on a BD FACSCanto II and cell populations determined using the BD FACSDiva software. Whole cells (as determined by the presence of an intact nucleus) were detected by nuclear staining, allowing the area of interest to be defined by forward and side scatters. Forward and side scatters were also used to gate areas for the detection of lymphocytes (T and B cells), natural killer cells (NK), macrophages (M0), and neutrophils.

### 2.4 Re-stimulation assay

Single-cell suspensions of spleen cells were diluted to achieve an approximate concentration of 1-3 × 10^6^ cells/mL and stimulated with either L-15 media (negative control), ConA (2.5 µg/mL positive control; Sigma, UK), or 1 × 10^7^ cfu/mL of heat-killed *F. tularensis*, for 18 h incubated at 37°C. The supernatant was removed and stored at −80°C prior to cytokine analysis.

### 2.5 Antibody ELISA

The levels of anti-*Francisella* antibodies (IgM and IgG) were measured in plasma which was stored frozen until analysis. MaxiSorb 96-well plates were coated overnight with 1×10^8^ cells/mL of heat-killed *F. tularensis* in carbonate coating buffer and blocked for 2 h with bovine serum albumin (BSA). Plasma, at a starting dilution of 1 in 50, was prepared for further dilution to 1 in 5 and was allowed to bind for 1 h. Antibody binding was detected with a goat anti-human IgG HRP (Sigma, UK) after a further hour.

### 2.6 Cytokine analysis

Cytokines and chemokines were measured in the plasma which were stored frozen at −80°C until required. Levels of both were quantified using the human BD cytokine flex beads to detect IL-1β, IL-6, MCP-1, and RANTES (BD Biosciences) and TNF-α and IFN−γ (reagents from U-CyTech and Mabtech AB, conjugated to flex beads by BBI Detection Ltd). All samples were fixed in 4% paraformaldehyde for 36 h at 4°C and analysed by flow cytometry (FACSCanto II BD Biosciences). Due to sensitivity issues, the quantification of IFN−γ was also determined using ELISA and the same antibodies (Mabtech AB).

### 2.7 Histopathological analysis

Tissues (lungs and mediastinal lymph nodes) were fixed in 10% neutral buffered formalin and processed for paraffin wax embedding using standard techniques. Thin sections (4 μm) were cut and stained with haematoxylin and eosin (H&E) for histopathological analysis or Gram Twort to allow for visualisation of bacteria. The tissues were examined by light microscopy and evaluated subjectively.

### 2.8 Immunohistochemistry

Sections of tissue were stained for immunohistochemical detection of *F. tularensis* LPS antigen (FB11), macrophages and neutrophils (MAC387), T cells (CD3), or B lymphocytes (CD79a). Sections used for immunolabelling were dewaxed and dehydrated, and any endogenous peroxidase activity was quenched in hydrogen peroxide 3% in methanol for 15 min to eliminate activity prior to antigen retrieval. The samples were treated with trypsin/alpha-chemotrypsin (0.5% trypsin and 0.5% alpha chemotrypsin; Sigma-Aldrich, Gillingham, Dorset, UK) at 37 °C for 10 min. Sections were then microwaved in citric acid buffer (2.1g citric acid; Fisher Scientific, Loughborough, Leicestershire, U.K., in 1,000 mL distilled water), pH 6.0 for 18 min, 90% power (780 W) or in Dako high pH 9.0 buffer (Dako UK Ltd) for 10 min. The sections were then assembled into Sequenza cover plates (Shandon Scientific, Runcorn, UK) and rinsed with Tris-buffered saline (TBS) pH 7.6, 0.005 M (Sigma–Aldrich, USA). Primary antibody cross-reactivity with tissue constituents was prevented by using a 1.5% goat serum block in TBS for 20 min followed by incubation with the primary antibody diluted in TBS for 1 h at room temperature. The sections were washed in TBS and incubated for 30 min with the Dako REAL EnVision™ polymer (Dako UK Ltd). The immunohistochemical signal was visualised using 3,3-diaminobenzidine (Sigma-Aldrich), and sections were counterstained in Mayer’s haematoxylin (Surgipath, UK), dehydrated in absolute alcohol, cleared in xylene, and mounted using dibutyl phthalate xylene (DPX) and glass coverslips. Positive and negative controls were used, and technique controls included serial sections incubated with a protein of the same isotype immunoglobulin for each primary antibody, and the omission of the primary antibody.

### 2.9 Statistics

Data from animals were grouped based on whether they succumbed to disease or survived treatment (regardless of the route of administration of ciprofloxacin). Where the timing of administration of therapy (i.e., Tx or PEP) affected the parameter being assessed (e.g., antibody level), the data is presented separately. All data is shown on the graphs with the median and interquartile range marked. Statistical significance was determined using a Kruskal–Wallis ANOVA or Student T test as appropriate (GraphPad Prism V10.0.3). As only one animal relapsed, data is presented for information and no statistical conclusions are drawn from this animal.

## 3 Results

### 3.1 Fever is associated with the increase in circulating neutrophils and IFN-γ as well as the activation of monocytes

**Figure 1:**
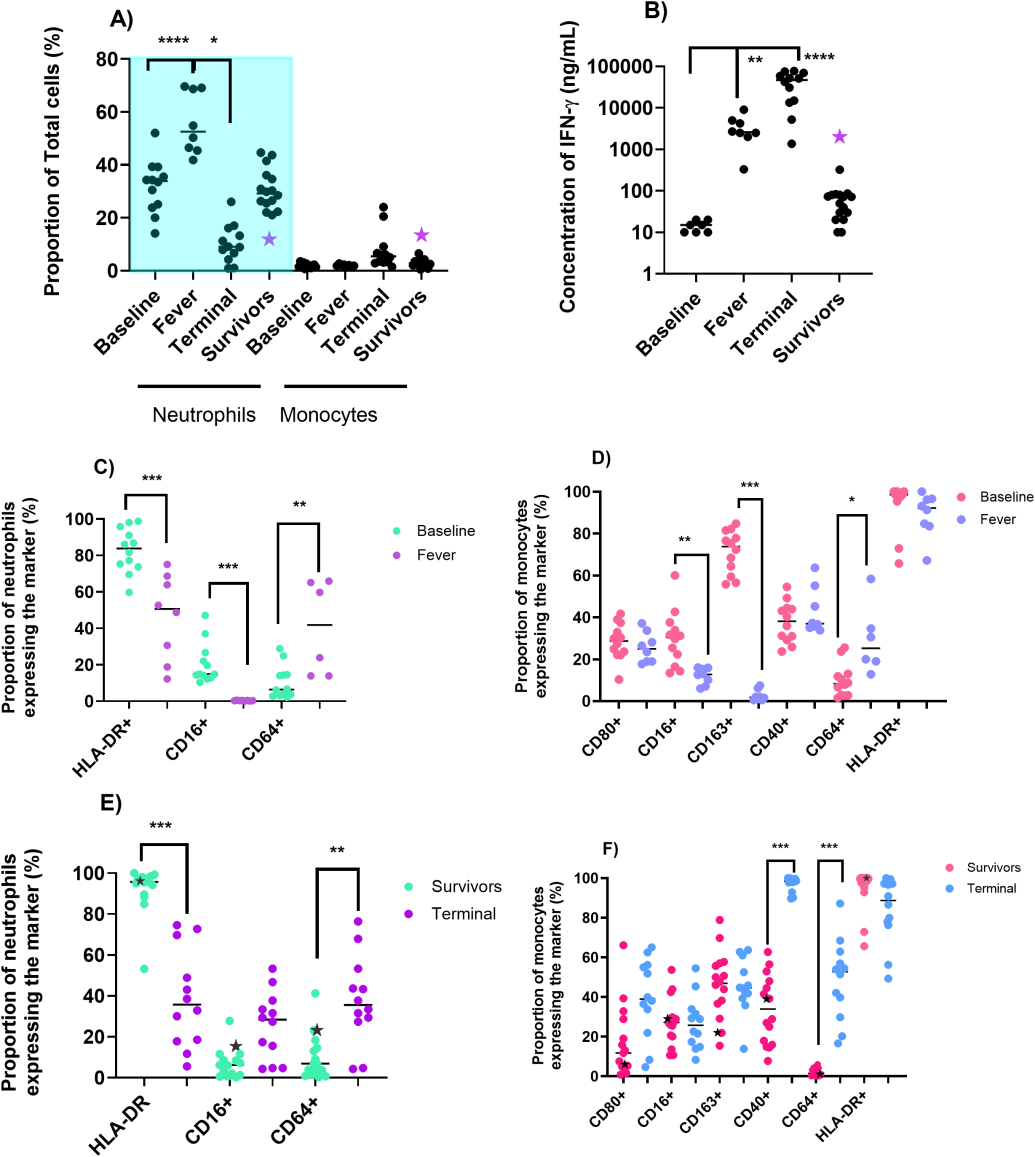
Cellular and cytokine changes associated with fever or terminal disease in animals challenged with *F. tularensis* by the inhalational route. **(A)** Proportions of circulating neutrophils or monocytes. **(B)** Levels of circulating IFN-γ. **(C)** and **(E)** Neutrophil activation marker expression. **(D, F)** Monocyte activation marker expression. Fever occurred on day 3 post-challenge, and animals succumbed to disease (terminal) on days 5-6. Data are presented as the median with interquartile ranges, significance by Kruskal–Wallis test **(A)** or Student T test *P<0.05, **P<0.01, ***p<0.001 and ****p<0.0001. The animal that relapsed is marked on the figures as a star, although the data point is not included in the statistical analysis. Note: some of the data in **Figure 1B** is included in (Ireland et al., 2026).

Blood collected from animals that had been challenged with *F. tularensis* and received ciprofloxacin by the oral or inhalational route was further analysed (Ireland et al., 2026). Levels of neutrophils, monocytes, and circulating IFN-γ were assessed at the onset of fever (between 57 and 70 h post-challenge) in animals that were untreated or received ciprofloxacin at the onset of fever (study Tx only). Compared to baseline, there was an increase in the proportion of circulating neutrophils (p<0.0001) and IFN-γ (p<0.01) but not the level of monocytes (**Figures 1A, B**). There was also a change in the phenotype, with a reduction in the expression of HLA-DR (p<0.001) and CD16 (p<0.001) and an increase in CD64 expression (P<0.01) (**Figure 1C**). Classical activation of the monocytes was observed with an increase in CD64 expression (p<0.05), and a reduction in CD16 (P<0.01) and CD163 (P<0.001) (**Figures 1C, D**). Levels of CD40 and CD80 remained relatively similar to baseline.

### 3.2 Terminal disease was associated with a strong innate immune response whereas the response in animals that recovered following treatment returned to baseline

Blood was collected from all animals at the time of euthanasia (either when they reached the humane endpoint between days 4 and 8 post-challenge or if they survived until the end of the study, days 21 to 24. Animals that received placebo (untreated) succumbed to infection and had a reduction in the proportion of circulating neutrophils in the blood (p<0.0001). The phenotype of the remaining neutrophils was comparable to those during fever, with a significant reduction in the expression of HLA-DR and an increase in CD64 expression (**Figure 1E**). However, the level of CD16 expression increased, particularly in comparison to the levels during fever. The proportion of monocytes increased slightly, especially in two animals; however, the expression of these markers suggested a classical activation status with ubiquitous expression of CD40 (p<0.001), increased CD64 (p<0.05), and a marginal increase of CD80 expression (**Figure 1F**). Circulating IFN-γ was greater than baseline levels (p<0.0001). Other circulating cytokines IL-6, IL-1β, MCP-1, and TNF-a were elevated above baseline (discussed in Ireland et al., 2026).

The circulating neutrophil and monocyte cell proportions and phenotypes in animals that were administered ciprofloxacin and survived returned to the normal range. However, the proportion of neutrophils in the lungs of 5 out of 12 of these animals were extremely high, which is characteristic of pneumonia. In the surviving animals, the levels of circulating cytokines had also returned to baseline, suggesting that these animals had completely recovered. One animal that received Apulmiq at the onset of fever relapsed with tularemia, i.e., developed a secondary infection and had a highly elevated level of monocytes and IFN-γ compared to the other surviving animals.

### 3.3 Prophylaxis limited the immune response whereas treatment on fever successfully triggered development of the adaptive response

**Figure 2:**
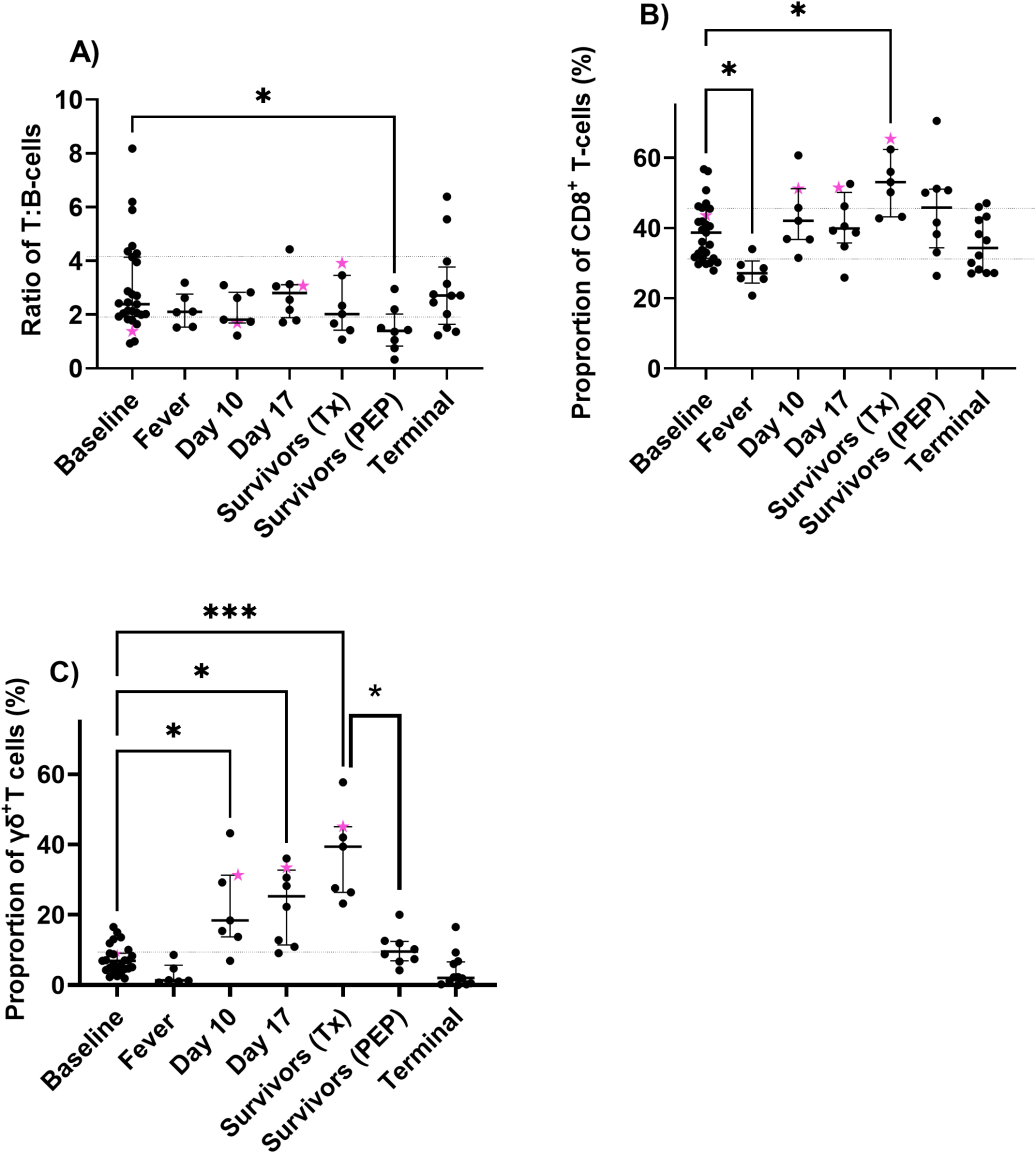
Changes in the activation state of circulating T cells in marmosets challenged with *F. tularensis* by the inhalational route and administered ciprofloxacin. The ratio of T to B cells **(A)**, and the proportion of T cells expressing CD8^+^ **(B)** or γδ+ **(C)** expression markers. Fever occurred on day 3 post-challenge, animals succumbed to disease (terminal) on days 5-6, treatment ended on day 10, in-life blood sampling occurred on day 17 post-challenge, and the study ended on day 21 (PEP) or day 24 post-challenge (treatment, Tx). The animal that relapsed is highlighted with a pink star. The significance from baseline values was determined by Kruskal–Wallis test where * P<0.05 and ***P<0.001.

The activation state of T cells was measured over time to determine whether there was a developing immune response. The ratio of T to B cells was determined to provide T-cell data, independent of the large fluctuations in neutrophil levels associated with fever or death (**Figure 2A**). This ratio was consistent over the course of the disease, with a minor increase in B cells relative to T cells in those animals given PEP. There was an increase in the proportions of CD8^+^ T cells, in the animals that survived following treatment at the onset of fever compared to baseline levels (p<0.05) or animals given PEP (not significant) (**Figure 2B**). This was associated with an expansion of the γδ-T cell population in animals treated at the onset of fever (Tx, p<0.001) compared to baseline and a difference in the levels between the two treatment groups (p<0.05) (**Figure 2C**). Expansion of the γδ-T cell population was significantly increased from day 10 post-challenge (p<0.05) for the treatment on fever (Tx) group and continued to increase over time (blood from the PEP group was not taken). However, it should be noted that approximately 50% of the γδ-T cells also expressed CD8 (a trend not related to treatment). In addition, there was no relationship between the levels of the T-cell activation markers CD69 or CD56 between the treatment groups.

**Figure 3:**
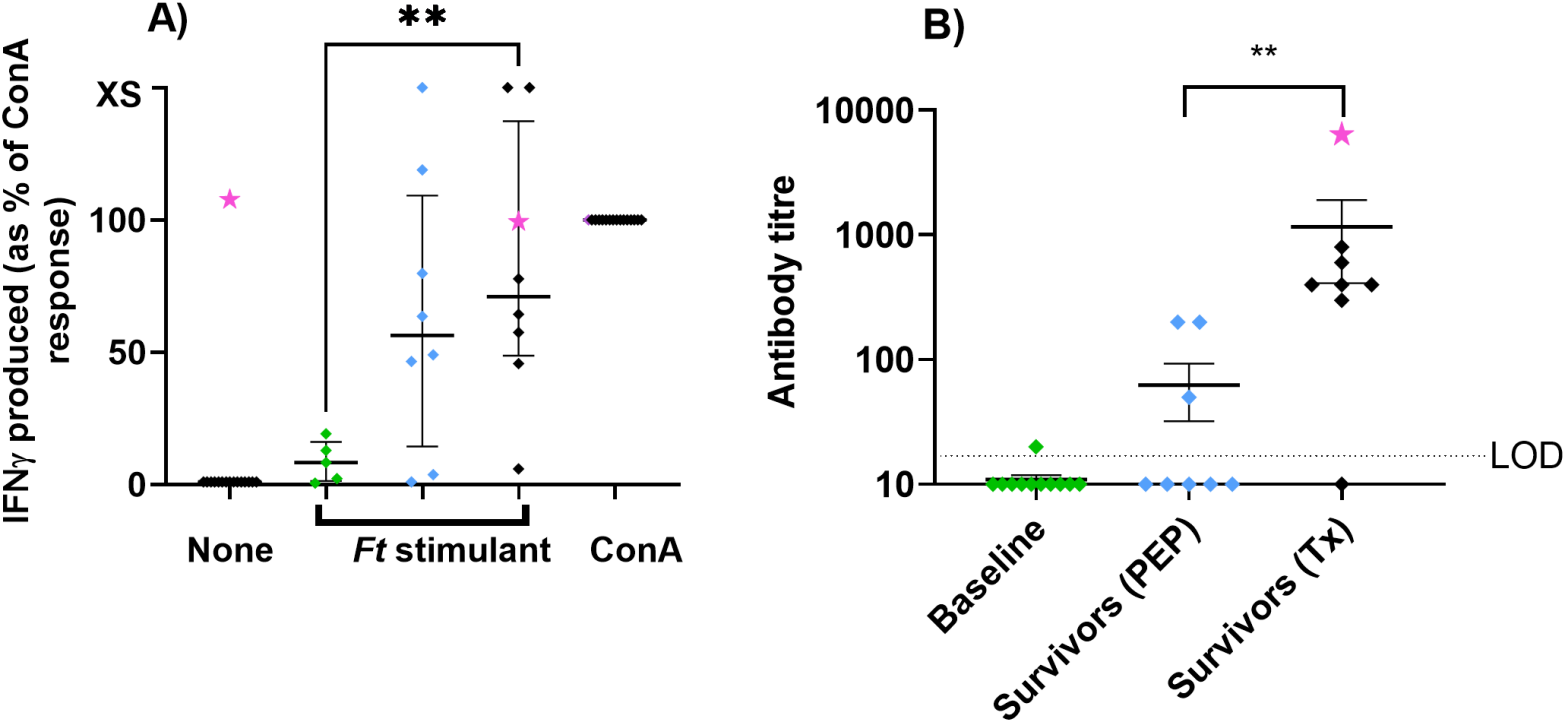
The immune response from the spleen cells and blood of animals challenged with *F. tularensis* and treated with antibiotics. The cell-mediated response **(A)** was assessed by the production of IFN-γ following a 24-h exposure of spleen homogenates to heat-killed whole-cell bacterial antigens. Naïve animals are shown in green, animals administered PEP are in blue, and animals treated at the onset of fever are in black. Concentration is expressed as a percentage of the total IFN-γ produced to the positive control, ConA. Circulating antibody (IgM) **(B)** was assessed from the same animals measured by ELISA. The animal that relapsed is highlighted with a pink star. Significance by Student t test **p<0.01. Note: an alternative version of **Figure 3B** is included in (Ireland et al., 2026).

To further assess the development of T-cell immunity, single-cell spleen homogenates were incubated with heat-killed *F. tularensis* to look for production of IFN-γ (**Figure 3A**). Animals challenged with *F. tularensis* produced more IFN-γ than naïve animals (p<0.01 for animals treated at the onset of fever). However, there was no difference in the response of animals treated at the onset of fever compared to those administered PEP. This suggests that the bacterial LPS was presented to T cells following infection, despite the animals administered PEP not showing signs of disease.

Circulating antibody levels were also assessed in animals that survived until the end of study (**Figure 3B**). No IgG was detected even at day 24 post-challenge. Higher levels of IgM were detected in animals treated at the onset of fever (p<0.01), with no antibody detected in five out of eight animals administered PEP.

### 3.4 One animal relapsed but demonstrated both innate and adaptive immune activity directed at combating disease

**Figure 4:**
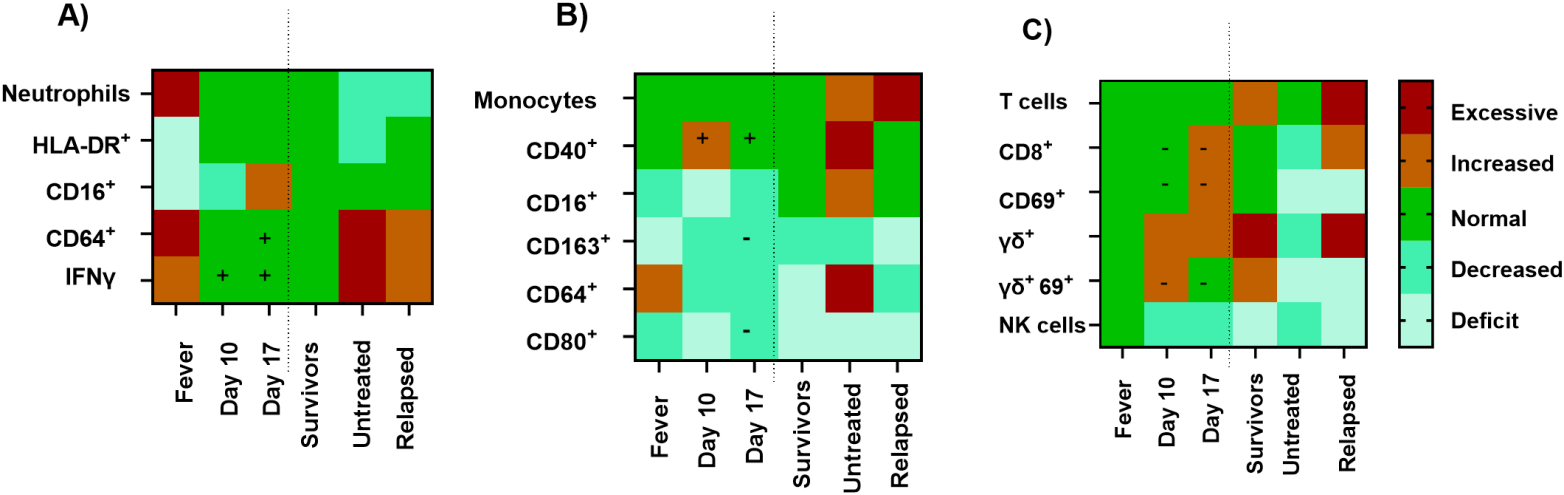
Heat maps to illustrate the changes in cell populations and activation markers in relationship to disease. **(A)** Neutrophils and IFN-γ, **(B)** monocytes, and **(C)** T cells. Values within the baseline range are green, with paler green colours indicating reduced levels compared to baseline, and orange to red indicating increased levels. The “+” or “–” superimposed on days 10 and 17 indicates that the levels of cells and markers in the animal that relapsed was higher (+) or lower (−) than the other treated animals, respectively. The fever data are a composite from all animals collected on day 3 post-challenge. The day 10 data are collected from animals at the end of treatment, and in-life blood collected from treated animals occurred on day 17 post-challenge. The remaining data were collected from animals at the time of euthanasia when they either succumbed to disease (untreated) on days 5-6, or at the study ended on day 24 post-challenge (Survivors and Relapsed).

In order to understand the possible prognosis for the animal that relapsed, the key immunological features of tularemia in marmosets were investigated further (**Figure 4**). The immunological pattern for the animal that relapsed is different from the other treated animals that survived but also from the untreated animals that succumbed to infection. On day 10 and day 17 post-challenge, there was elevated IFN-γ and an increase in CD40 expression on macrophages (indicating classical activation) in the animal that relapsed compared to the other treated animals. This suggests an immunological battle in an attempt to control the infection. The amount of circulating IFN-γ detected (3,000 ng/mL at the end of treatment on day 10) was comparable to the levels detected in animals during the initial fever (mean of 4,250 ng/mL), rather than the extremely high levels associated with terminal disease (>10,000 ng/mL). The levels of other cytokines were not assessed at this time. By the end of the study (day 24), the population of macrophages had expanded beyond normal limits for this animal only. These were 13% of the total peripheral blood mononuclear cells (PBMCs) compared to 2% for the other treated animals and 6% for the control animals. However, the level of circulating IFN-γ had reduced to 2,000 ng/mL and the activation status of the macrophages was comparable to the animals that had survived following treatment. The animal that relapsed had clear indications of developing adaptive immunity including high proportions of T cells, CD8^+^ T cells, and γδ^+^ T-cells, in addition to high levels of antibody.

**Figure 5:**
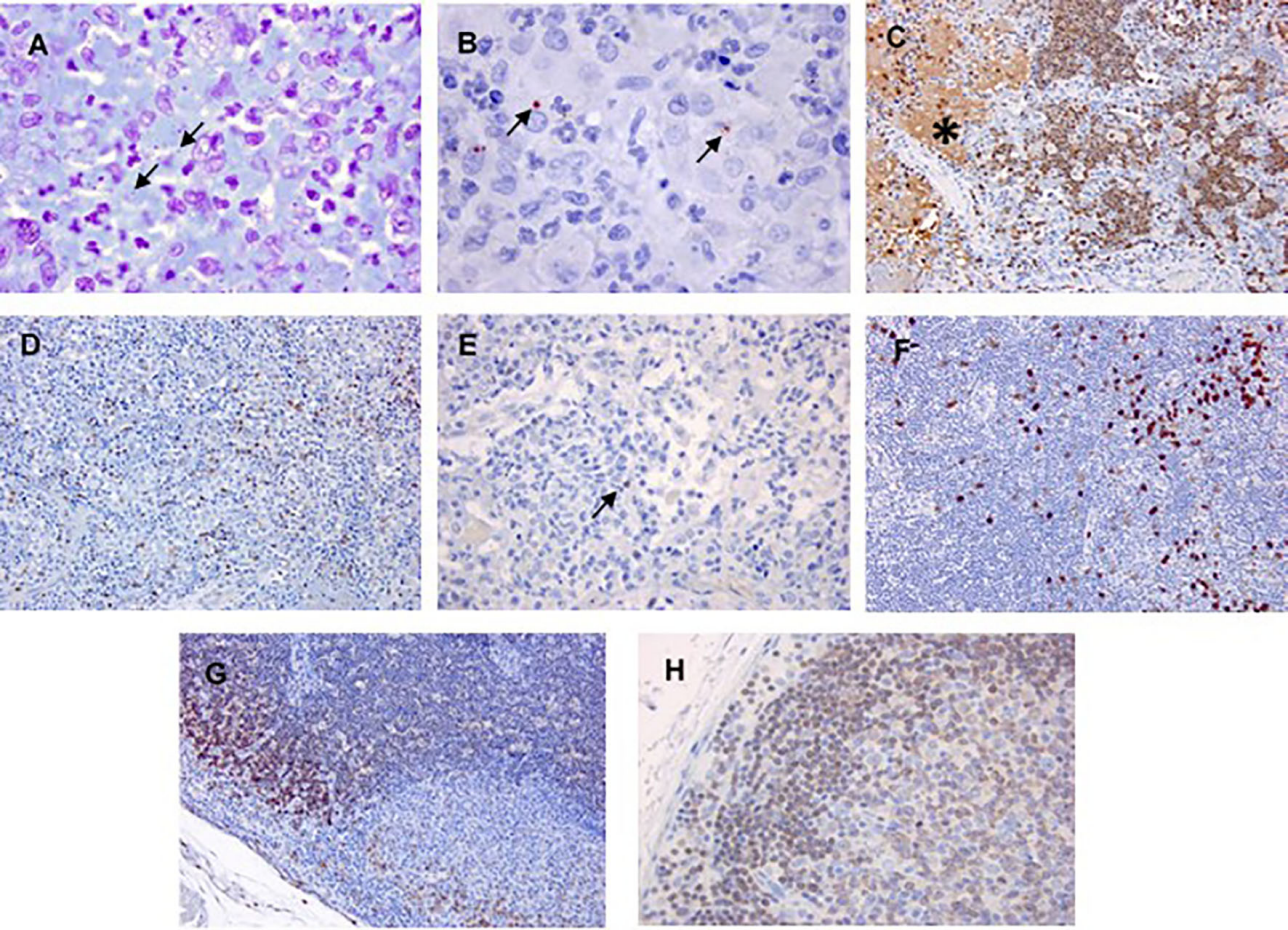
Representative images of the lungs and mediastinal lymph node from the animal that relapsed following challenge by the inhalational route with *F. tularensis* and treatment with Apulmiq. Sections of the formalin fixed lungs were processed, stained with Gram Twort, or treated with a panel of antibodies. **(A)** Lung section with Gram Twort staining resulted in the visualisation of small Gram-negative coccobacilli in acute pulmonary lesions (1,000× magnification). **(B)** Lung section of *F. tularensis* bacterial cells labelled with FB11 (1,000× magnification). **(C)** Lung section stained with MAC387 detected neutrophils and macrophages in areas of acute inflammation. Extracellular labelling was also observed, likely due to presence of cellular components and debris in the exudate (200× magnification). **(D)** Lung section stained with CD3-positive cells were numerous in the acute exudation (200× magnification). **(E)** Lung sections stained with CD79a-labelled B lymphocytes were detected in areas of acute exudation (400× magnification). **(F)** MAC387-labelled macrophages in the cortex (200× magnification). **(G)** Mediastinal lymph node section with CD3-positive cells in the cortex (200× magnification). **(H)** Mediastinal lymph node section with CD79a-labelled B lymphocytes in the cortex (400× magnification). * - areas of diffuse extracellular labelling. The arrows in **(A, B, E)** are highlighting individual bacterial cells. Bacteria in Image B were shown to be viable in Ireland et al. (2026). Images **(B, C, G)** were also included in Ireland et al. (2026).

Gram Twort and imummohistochemical (IHC) staining of lung tissue from the animal that relapsed shows the presence of bacteria (**Figures 5A, B**). This was not evident in other tissues from this or any other animal that were treated with antibiotics. Neutrophil infiltrate was also evident in the lung tissue aligning with the flow cytometry data (**Figure 5C**). This neutrophil infiltration was also observed in untreated, but not other treated animals. Small numbers of T and B-cells were also observed (**Figures 5D, E**). Cellular infiltration was also apparent in the mediastinal lymph nodes from the animal that relapsed (**Figures 5F–H**).

## 4 Discussion

The aim of this work was to descriptively elucidate the immunological response in infected marmosets to determine whether any key immunological features could be associated with effective treatment, survival, or potential relapse of disease. The immunological profile demonstrates how similar the lethal disease observed in marmosets is with that of humans. In humans, *F. tularensis* primarily infects macrophages (Fortier et al., 1994; Roberts et al., 2014; Fortier et al., 1992). This work demonstrated how macrophages were heavily involved in the marmoset immunological response to infection. This is different from what is observed in other marmoset models of bacterial infection, including melioidosis, where neutrophils play a much bigger role (Ngugi et al., 2022). Neutrophil recruitment into the lungs is important in pneumonic tularemia, but if extensive can cause considerable damage (Conlan et al., 2003; Malik et al., 2007). This was clearly observed in 5 out of 12 animals that were not treated with antibiotics following challenge *with F. tularensis* (Ireland et al., 2026). The disease is generally characterised by bacterial uptake into cells and a poor immunological response, enabling initial bacterial replication inside the macrophages, eventually producing IFN-γ and the classical activation of macrophages (Fortier et al., 1992, 1994; Andersson et al., 2006). This activation should be sufficient to kill *F. tularensis*; however the uncontrolled bacterial growth that occurs before the cytokine response is often overwhelming. The detection of infection and IFN-γ production occurs too late to prevent systemic dissemination and sepsis (Fortier et al., 1992; Elkins et al., 2007). This aligns with the key features described in the marmosets; high levels of IFN-γ were produced when the animals were in fever (day 3), which led to an increase in the proportion and the classical activation of the circulating monocytes. However, this response was insufficient to protect animals that were not treated with antibiotics.

The immunological response in the marmoset was different between healthy and infected animals. The early increase in the proportion of circulating neutrophils at the onset of fever has been identified as characteristic in other NHP models (Glynn et al., 2015), although the reduction of neutrophil expression of HLA-DR is unique to marmoset infection models (Ngugi et al., 2022). Fever was also associated with elevated circulating IFN-γ. Recovery from infection in the marmoset was suggested by the level and activation state of neutrophils returning to those observed in healthy marmosets combined with increased activation of CD40 but not elevation in macrophage proportion, and normal levels of circulating IFN-γ. This, combined with a return to normal body temperature (and behaviour), provides confidence of a complete recovery.

The T-cell changes, including the IFN-γ production in response to re-stimulation, observed between paired blood samples (baseline and post-treatment), was suggestive of the development of adaptive immunity in the marmoset. However, this study did not unequivocally demonstrate that the increased T-cell activity was a correlate of protection. Vaccine studies in mice have shown that both CD4^+^ and CD8^+^ T-cells are required for protection (Conlan et al., 1994; Roberts et al., 2014; Mara-Koosham et al., 2011). Human T cells respond by proliferating and secreting cytokines, predominantly IFN-γ, when exposed to *F. tularensis* antigens after naturally occurring infection or vaccination (Elkins et al., 2007; Lindgren et al., 2023; Salerno-Gonçalves et al., 2009).

The role that γδ T-cells (specifically the V9γV2δ subpopulation) play in human disease is unclear, but their proliferation during human tularemia has been reported (Sumida et al., 1992; Poquet et al., 1998). Marmosets also have this specific subset of T cells, a subset which small rodents cannot produce (Rowland et al., 2012). Recently, it has been shown that the increase in γδ T-cells in human tularemia can be measured using any pan γδ T-cell marker (Chrdle et al., 2019) and the marker used in this work has been successfully used to monitor γδ T-cell activity in other human diseases (Jenjaroen et al., 2015). The relationship between γδ T-cells and tularemia is apparent in the marmosets, with a significant increase in the levels over time. The relevance of γδ T-cell controlling the disease in humans has been discussed because the increase occurs slowly, observed from day 8 of the infection onwards, peaking by day 21 to a maximum of 50% of all T cells (Kroca et al., 2000, 2001). This correlates well with the marmoset data with evidence of activation (CD69^+^) on day 10, at the end of treatment. In countries including the Czech Republic, an increase in pan γδ T-cells or CD4^−^/CD8^−^ T-cells is now recognised an early presumptive diagnosis bringing this forward by 7–10 days (Chrdle et al., 2019). It would have been interesting to isolate and culture these marmoset cells to determine if they had the ability to control *F. tularensis* growth within a cell-based assay (Bosio and Elkins, 2001; Rowland et al., 2012).

It has not been confirmed as to whether the marmosets had developed a protective immune response, but the IFN-γ production observed in a cell restimulation assay was encouraging. This aligns with the measurable IFN-γ response observed as early as 10 days post-fever in treated human cases (Eliasson et al., 2008). However, there was no IgG response in the marmosets in contrast to humans where both IgM and IgG are detectable in 90% of human cases by day 21 (Eliasson et al., 2008; Maurin, 2020). In fact, other studies found that an antibody titre high enough to act as a diagnostic marker (titre 1:20) was detectable around 30 days following the development of symptoms. However, administration of antibiotics may delay or suppress measurable antibody. The role of antibody in the protection against virulent disease is still much debated, but any protection afforded by antibody relies on intact and effective T-cells (Mara-Koosham et al., 2011). The difference could be related to the stimulating antigens as the human response is almost entirely directed towards LPS, whereas other animal species (especially mice, but also some NHPs) are known to be more tolerant of LPS (Rahhal et al., 2007; Munford, 2010). The predominant target of the marmoset antibodies was not elucidated, but determining that would be relevant for any vaccine studies.

For the marmoset that relapsed, the key immunological characteristics suggested that the infection was being controlled and the changes measured over the timeframe of the study suggests a slow improvement. It is unclear however whether the animal could have maintained the immunological response sufficiently to have cleared the infection or whether there was too much damage to the lungs for the animal to have recovered. For humans, a second course of antibiotics and some supportive therapy would have been prescribed, and recovery would likely have been achieved (Bahuaud et al., 2021). The elevated level of circulating IFN-γ detected in the marmoset that relapsed, at the end of treatment, was the first indication of a different response and highlights the importance of IFN-γ. IFN-γ is known to play a pivotal role in human tularemia (Andersson et al., 2006; Elkins et al., 2007), and its detection is key to understanding the health status of subjects in infectious disease models. Detection of IFN-γ was not fully investigated in the cynomolgus model of tularemia (Frick et al., 2021; Glynn et al., 2015; Guina et al., 2018). However, production of IFN-γ following re-exposure in *ex vivo* cell-based assays from vaccinated or infected/recovered individuals is the best correlate of protection identified to date (Eneslätt et al., 2018).

In conclusion, this work highlights that characterisation of the immunological response can enrich the data generated in these models and aid with interpretation. Terminal disease was associated with sustained innate immune activation, whilst recovery was correlated with the normalisation of immune parameters. Treatment at fever onset, but not prophylactically, led to expansion of CD8^+^ and γδ T cells, and increased IFN-γ production upon antigen re-stimulation, suggesting the development of adaptive immunity. The animal that relapsed post-treatment, exhibited elevated IFN-γ and monocyte activation, in addition to colonising *F. tularensis* in lung tissue, suggesting an ongoing immune response insufficient for clearance. These findings demonstrate that marmosets exhibit immunological responses closely mimic human tularemia, including key innate and adaptive features. The model provides valuable insight into disease progression, treatment efficacy, and relapse, supporting its utility for evaluating medical countermeasures.

## Data Availability

The original contributions presented in this study are included in the article. Further inquiries can be directed to the corresponding author. Requests to access the datasets should be directed to mnelson@dstl.gov.uk.
